# Voices from the ground: Qualitative perspectives on Ugandan national mental health system strengths, challenges, and recommendations

**DOI:** 10.1371/journal.pmen.0000077

**Published:** 2024-11-12

**Authors:** Lekie Dwanyen, Ibrahim Luberenga, Ronald Asiimwe, Pendo Galukande, Adrian Blow, Rosco Kasujja

**Affiliations:** 1 Michigan State University, East Lansing, Michigan, United States of America; 2 University of Minnesota, Minneapolis, Minnesota, United States of America; 3 Makerere University, Kampala, Uganda; James Cook University - Singapore Campus, SINGAPORE

## Abstract

Mental health capacity building is a growing priority in Ugandan healthcare systems. Despite increased governmental attention to community mental health and mental healthcare needs, no empirical assessments document qualitative perspectives from stakeholders in the Ugandan mental health system. The goal of the current needs assessment study was to systematically explore stakeholders’ perceptions of strengths, challenges, and recommendations for enhancing capacity in the national mental health system in Uganda. Using ethnographic research methods, data were collected from 15 key informant interviews and four community focus groups with a total of 44 stakeholders involved in mental healthcare in Uganda. Thematic analyses of data yielded several themes in each domain, including 1) system strengths reflected in the *existing policy and action plan*, *free medications*, *growing private sector*, *and partnerships and collaborations; 2)* system challenges including *socioeconomic constraints*, *stigma*, *and limited family engagement;* and 3) recommendations surrounding *enhanced education and training*, *integrating systemic approaches*, *and policy advocacy*. We describe clinical, research, and policy implications that can inform systemic mental health efforts in Uganda and comparable global settings.

## Introduction

Uganda is relatively progressive among African nations in its establishment of mental health legislation, and growing research and practice sectors [1]. The 2019 National Mental Health Action Plan increased attention to mental health legislation, practice, research, and other priorities to address significant mental health needs across Uganda. The action plan increased awareness of non-medical psychosocial professionals’ (e.g., psychologists, counselors, social workers) contributions to mental healthcare in Uganda. Despite progress, the Ugandan mental health system continues to experience burden from limited resources, both in terms of finances and human capital, to deliver quality mental healthcare to the expanding population. To illustrate, only one percent of Uganda’s annual healthcare budget is allocated to mental health [2], proving insufficient to address escalating mental healthcare demands within the growing population. The global refugee crisis amplifies the need for national mental health responses [3]. As one of the top-receiving nations for refugees in Africa and globally, the Ugandan mental health system is in critical need of capacity building efforts.

While progress in mental health research, practice, and policymaking is expanding [2], a dearth of research highlights the experiences and insider perspectives of key stakeholders involved in mental healthcare in Uganda. The goal of this study was to understand diverse stakeholders’ (e.g., psychiatrists, clinical and counseling psychologists, graduate trainees, service users, policy officials) perceptions of strengths and challenges within the Ugandan mental health system, and recommendations to enhance capacity, particularly to support common family- and community-level mental health needs.

## Literature review

### Global mental health

The World Health Organization (WHO) estimates that approximately one billion people are living with mental health disorders globally, with 75% of the global disease burden affecting lower- middle-income countries (LMICs) [4]. Global mental health aims to improve access to quality mental health services in diverse settings globally [5,6]. Global mental health includes research, training, and practice rooted in social justice principles surrounding equity, access, community, culture, and context [7]. The Center for Global Mental Health Research by The National Institute of Mental Health (NIMH) recognizes that improving the availability, quality, and accessibility of culturally appropriate mental health resources in LMICs is imperative to improving mental health outcomes across global settings [8,9].

Mental health challenges persist in sub-Saharan Africa due to inadequate access to quality mental health care in most countries. Studies show that the global average of mental health professionals per service user is 9.0 per 100,000 service users, however, in the SSA region, there are only 1.4 mental health professionals per 100,000 service users [10]. Moreover, estimates show that the population of Africa is expected to double over the next three decades, implying that the rates of mental health needs and disparities are also likely to increase [11]. Particularly, mental health of African youth is likely to be jeopardized by the highly competitive labor market, and as many fail to achieve their economic ambitions, some are likely to turn to substances (e.g., alcohol, marijuana, etc.) to cope with their frustrations.

### Mental health in Uganda

Mental health and substance use disorders represent major public health concerns in Uganda [12]. Depression, anxiety disorders, and chronic stress are common; Uganda ranks among the top six African countries for rates of depressive disorders [13]. After two decades of civil conflict in northern Uganda, post-traumatic stress disorder (PTSD) rates as high as 54% are documented in certain communities in the region [12]. Research also reveals high rates of intimate partner violence [14] and disruptions in parent-child relationships [15]. Like elsewhere, social, and economic losses (e.g., isolation, unemployment) from the COVID-19 pandemic exacerbated mental and relational health concerns, and the rates of depression, anxiety, posttraumatic stress, and substance use disorders are projected to continue rising in Uganda [16]. Uganda is also one of the top-receiving African nations for refugees and asylum seekers. More than 1.6 million refugee individuals and 400,000 refugee and asylum-seeking households were recorded in Uganda at the end of 2023 [17]. This influx amplifies the need for quality and accessible mental healthcare in Uganda.

#### Background on Ugandan mental health system

Until the late 1990s and beginning of the 2000s, mental health services in Uganda received minimal attention and limited national funding. The Ugandan government established frameworks for policies and strategies to direct the delivery of mental health care around the beginning of the twenty-first century [18]. These included the Mental Health Act of 1964, which updated in 2006 to reflect contemporary norms, protect the civil rights of people with living with disabilities, and ultimately provide inclusive and integrative mental health care in Uganda. Despite these policies, Ugandan mental health settings continue to be underfunded, with one percent of the annual national healthcare budget allocated to mental healthcare [2]. Butabika National Referral Hospital, Uganda’s primary center for mental health treatment, offers inpatient and outpatient services, community outreach, counseling, and rehabilitation programs [19]. Staff at Butabika face difficulties due to lack of funding and limited human resources [20].

#### Ugandan mental health workers

Inadequate government funding limits employment of qualified mental health providers in the national mental health system [21]. Psychiatry is the only discipline officially employed by the government. However, only 32 psychiatrists and 60 psychiatric clinical officers support psychiatric services across the country [22]. There are more than three times the number of clinical psychologists, counseling psychologists, social workers, and counselors in-country, most of whom are employed in the non-governmental (NGO) and private sectors. Traditional healers also play a key role in Uganda’s mental health landscape [23]. Studies show that up to 60% of Ugandans seek support from traditional healers, as well as spiritual and religious leaders, to cope with personal health and relationship issues [22,24]. Despite popularity, traditional, spiritual, and religious leaders often have limited training in evidence-based mental healthcare [25,26]. Investment in training mental health professionals; integrating traditional, spiritual, and religious leaders; expanding mental healthcare to marginalized areas; and funding mental health research are all necessary for building capacity [27].

## The current study

The purpose of this study was to conduct a local needs assessment of the national mental health system in Uganda. This study contributes to the limited but growing body of literature published on global mental health capacity building from the direct perspectives of mental health workers and stakeholders. In addition, we explored participants’ perceptions of common help-seeking experiences in families. In the current paper, we detail findings related to stakeholders’ perspectives on system-level strengths, challenges, and recommendations for building capacity to respond to common family and community mental health problems in Ugandan settings.

### Researcher positionalities

This research took place in the context of an academic partnership between researchers at a major public university in the U.S. Midwest and the largest higher education institution in Uganda. The partnership was born out of authors’ shared interests around: 1) global mental health research, 2) mental health equity in Africa, and 3) access to culturally-grounded systemic interventions for families in Uganda. This partnership and the many alike are important to global mental health efforts as we conduct this research to ultimately improve access to quality mental healthcare for populations in lower-income settings, such as those in Sub-Saharan Africa [28]. Collaborations between scholars from high-income and lower-income settings ensures bolsters cross-cultural research capacity and opportunities for sustainable change. Together, research team diversity and our shared interests around mental health equity in Africa added value, direction, and richness to this study.

## Methods

### Study design

Ethnographic research methods informed the design of this qualitative needs assessment to honor the central influence of culturally- and contextually-grounded perspectives in the current study with Ugandan mental health stakeholders. Ethnographies are well suited for in-depth analyses of everyday lived experiences and culturally nuanced social processes in the natural setting of a culture-sharing group [29]. An ethnographic design aligned with our shared goals to garner deeper understandings of mental healthcare in Uganda, while attending to cultural and contextual assets in stakeholder settings (professional/clinical, educational/training, etc.). The Developmental Research Sequence [30]–a 12-step methodological guide for conducting and analyzing ethnographic interviews guided aspects of the current study design and research processes to examine: 1) system strengths, 2) system challenges, 3) system recommendations, and 4) youth and family-level mental health experiences from the perspectives of mental healthcare stakeholders in Uganda.

### Recruitment and sampling

Snowball sampling methods were used to recruit 44 stakeholders, including key informants (n = 15) and focus group participants (n = 29). We began by contacting an initial list of institutional leaders at Butabika National Referral Hospital, Makerere Schools of Medicine and Psychology, and the Ministry of Health. Key informants identified as clinical psychologists (n = 8), psychiatrists or psychiatric clinical officers (n = 5), a traditional healer (n = 1), or a public health officer (n = 1). Eleven key informants were interviewed in central Uganda, and four key informants were interviewed in northern Uganda.

Focus groups helped generate dialogue between stakeholders working in shared settings at the time of the study. Three groups were held in central Uganda, and one was held in northern Uganda. Central region focus groups included psychology graduate trainees (n = 7), practitioners at a local counseling center (n = 5), and service users (n = 9). In northern Uganda, one focus group was held with service users (n = 8). Across the 44 participants, an equal number of stakeholders identified as women (n = 22) and men (n = 22).

### Data collection procedures

This study was approved by the institutional review boards (IRBs) at the higher education institution employing U.S. team members and the higher education institution employing team members in Uganda. Participants provided written consent to audio record interviews for later transcription and analyses. The informed consent process also clarified stakeholders’ voluntary participation, and their rights to privacy and confidentiality, including de-identification in reports of study results (data available upon request). Key informant interviews lasted one hour, and focus groups lasted 90–120 minutes; both were semi-structured. The first grand tour question [30] explored participants’ roles and how they became involved in the mental health system. We asked, “As a [e.g., psychiatrist, psychologist, graduate student, institutional leader, service user], how do you typically engage with the national mental health system?” What is an ordinary day like in your role?” This was followed by mini tour questions in three domains to understand their views on: 1) strengths, 2) challenges, and 3) recommendations for building capacities to address common family and community mental health challenges in Uganda.

### Data analysis

Following data collection, the research team developed and reviewed field notes, memos, and initial summaries of key perspectives from the data collection phase. This early processing as a full team enabled reflexivity and analytic dialogues prior to in-depth analysis of the transcripts. Interview and focus group recordings were transcribed by local Ugandan graduate students, supporting a key goal to develop transcripts as close to local idioms and expressions as possible. Taken together, initial summaries were included with interview and focus group transcripts to enable triangulation during analyses. Data were analyzed using thematic analysis [31] strategies to develop categories, themes, and sub-themes representing findings from the needs assessment. Three research team members equally divided and analyzed one third of transcripts using thematic analyses procedures in full-text deductive analyses of participants’ descriptions of strengths, challenges, and recommendations reported herein.

### Enhancing trustworthiness

The research team used several methods to ensure quality and rigorous data collection and data analysis procedures, as well as the representation of results. Lincoln and Guba’s [32] trustworthiness criteria of *credibility*, *dependability*, *confirmability*, and *transferability* were used to guide and strengthen the rigor of the current study as these criteria are well established in the qualitative research literature [32]. Credibility, which reflects the degree to which findings accurately reflect the lived realities and experiences shared by participants, was enhanced by prolonged engagement, persistent observation, and data triangulation strategies.

Our research team is comprised of African mental health clinical researchers, several of which are natives of Uganda. As a credibility strategy, prolonged engagement helped us to build awareness of the study context and rapport with stakeholders. The research team spent nearly one year planning the study prior to data collection. Reflexive dialogues were held via Zoom starting in early phases to process personal values, biases, and assumptions that influenced study goals and subsequent data collection and analysis. Persistent observation, or the repeated observation of relevant study contexts, was also discussed as a strength among local team members as their knowledge and engagement in various mental healthcare settings in Uganda strengthened participant recruitment and sampling efforts, as well as contextually and culturally relevant interpretations of the results. Data triangulation, or the use of multiple types of data (i.e., interview and focus group transcripts, audio recordings, field notes, memos) were used to enhance analyses, emerging interpretations, and overall study rigor.

To enhance *dependability* or confidence in the study findings across time, researchers consistently documented key approaches, decisions, and techniques employed for data collection and analyses. We developed a comprehensive log for data management and analysis, which enabled an external audit to be conducted by a team member who was not involved in data collection and analysis procedures. This audit trail and documentation of study procedures enables reproduction of the study and enhances the dependability of the study results. Providing thick descriptions of data is a strategy that enhances *transferability* of research studies. This strategy is reflected in the results section, which includes rich, in-depth participant quotes to represent results as close to participants’ perspectives and observations as possible. We include both brief and detailed quotes to demonstrate key findings.

## Results

The results section details participants’ reflections thematically and includes in-depth quotes to illustrate stakeholders’ insights on strengths, challenges, and recommendations for enhancing services, training, and supervision capacity in national mental healthcare efforts in Uganda. Table 1 provides an overview of key themes and sub-themes related to study results.

**Table 1:** Overview of key domains, themes, and sub-themes.

Domain	Theme
**System Strengths**	Existing Policy and Action Plan
	Free Medications
	Growing Private Sector
	Partnerships and Collaborations
**System Challenges**	Socioeconomic Constraints
	Community and Professional Stigma
	Limited Family Engagement
**System Recommendations**	Enhance Education and Training
	Integrate Systemic Approaches
	Policy Advocacy

### Strengths of the mental health system

Participants described a range of strengths, assets, and resources that promote current mental health service delivery, training, and supervision of services to address common family and community mental health needs in the Ugandan mental health system. Reported strengths surround the existing mental health policy, free psychotropic medications, the availability of a growing private sector, partnerships and collaborations, and the use of evidence-based interventions to address community mental health needs.

#### Existing policy and action plan

Most participants acknowledged that there is a mental health policy that exists in Uganda to support human resources and professionals working in national mental healthcare. There is not only a government vision for mental healthcare but also current momentum as policies are being implemented. The government acknowledges mental health as a public health issue at various community levels. As one key informant shared, “…*since 1990s it [mental health] has been taken on as a minimum in [the] healthcare package that had to be integrated at the primary health level*, *so now we are getting into 30 years of that*…” (K8). Another stakeholder discussed the goal for policy, stating “*we are actually*… *revising the clinical guidelines and we are trying to see that we include non-medical treatment as part of treatment in mental health*.”

#### Free medications

Key informants widely agreed that the availability of free psychotropic medications is one of the greatest strengths and resources available in the mental health system. Most key informants discussed this as an asset in the national mental health system because individuals are not required to pay for medication they are prescribed by a psychiatrist. A sentiment shared by many, one stakeholder shared “*We have also improved our medicines list…generally the medicines are there*, *the people who are to give them out are also there*, *you need to see a psychiatrist*, *you don’t pay* (K2).” Most medications are delivered from India, linking to the strength in partnership between the Ugandan national mental health and Indian health and pharmaceutical systems. Key informants shared that medication supplies sometimes run out, presenting a challenge for medication access and management. However, free medications were framed as a strength given their lack of cost, which would have otherwise been prohibitive.

#### Growing private sector

Participants discussed ways that clinical and counseling psychology have grown in recent decades and resulted in trained professionals with the knowledge and capacity to engage in psychosocial efforts. Although this strength is limited by lack of funding to employ many of these trained individuals in the national mental health system, both central and northern Uganda were described as contexts with robust private sectors supported by professionals trained in psychosocial mental health approaches. As one key informant discussed their perceptions of human resources availability and training of community mental health workers, *“We have the human resources*, *not so specialized but at least they can offer the basics like managing the common mental health disorders at the community level”* (K8). This available network means there are professionals ready to receive additional training and support to integrate family and community mental health models in both private sector (e.g., NGOs) and government-funded settings (e.g., Butabika hospital, district-level hospitals) provided the opportunity for employment in national mental healthcare efforts. A majority of clinical psychologists, counseling psychologists, counselors, and other psychosocial workers are employed in private sector settings, and this private sector workforce plays a crucial role in offering supportive services, increasing awareness, and reducing mental health stigma.

#### Partnerships and collaborations

Key informants identified clinical intervention and research collaborations as important sources of community mental health support in Uganda. Several key informants shared about the positive impact of clinical research studies on local mental health efforts across Uganda. Research collaborations were noted as key to advancing knowledge and improving existing clinical strategies, particularly when developed in partnership with local researchers, clinicians, and community members. Participants credited collaborations for existing progress in evidence-based intervention science and delivery. Cognitive Behavioral Therapy (CBT) and Group Interpersonal Therapy (IPT-G) for depression were noted as the most common interventions observed and endorsed by stakeholders. As one informant stated when asked about contextual fit, *“…a lot of IPT-G*….*which is specializing in treating depression*. *And CBT*. *Where I’ve seen it work is within the humanitarian setting”* (K6). A few stakeholders acknowledged that although limited, growing attention to family-level interventions reflects progress in mental health efforts. As one recounted, “*[NGOs] are testing play therapy and it is implemented within schools… they go and assess those who might be having issues*, *those who might be not performing very well maybe they have problems homes and so they take on these play therapy which helps them think about whatever they are going through*….*”* (K8). Participants discussed their awareness of family interventions that were tested to reduce corporal punishment, youth conduct problems, and other child and family-level issues.

### Challenges in the mental health system

Key informants shared perspectives on a range of challenges and barriers to family and community mental health, most of which were explained by themes of economic constraints, stigma, and burden on the psychiatric care system/limitations around family-based intervention. Economic constraints and stigma were discussed across participants as multi-level concerns impeding existing mental healthcare efforts. The existing burden on the psychiatric system also limits availability and capacity to integrate family-based mental health practices.

#### Socioeconomic constraints

Participants identified socioeconomic constraints as an enduring hindrance to mental healthcare, including strains in institutional funding and family economics. Family financial and economic difficulties were largely attributed to Uganda’s status as an LMIC, as articulated by one participant, *“this being a low-middle-income country*, *I think priority goes to survival”* (K5). This sentiment reflects what numerous stakeholders acknowledged; for many Ugandans, supporting numerous family members’ needs with limited financial resources substantially constrains the ability to financially support mental healthcare for one or more family members.

#### Community and professional stigma

Stigma was discussed as a multi-level barrier. Negative attitudes toward mental health not only emerge in families and communities, but also permeate professional settings. Cultural stigma in families, communities, and professions operate to hinder mental healthcare. Participants described their observations of stigma at both family and community levels citing factors such as limited knowledge, fear, and reluctance to engage in discussions about mental health and the needs of family members. As one participant openly expressed, *“many people fear the diagnosis of a mental illness*. *They will accept to go to a health facility for anything but not for a mental illness* (K7).” Family and community stigma stem from limited mental health knowledge and education, and negative stereotypes around help-seeking. As one participant illustrated, *“attitudes towards mental health services and the changes our youth are experiencing*, *it is not a good one*, *it’s not good*. *Because in Uganda*, *when or if the youth is found abusing substances*, *in most cases they treat it as their own fault*. *We treat it as a lifestyle choice that you made*, *and you [youth] should be able to face the consequences (K12)*.” They described typical consequences to illustrate stigma, *“That is the attitude out there*, *in fact*, *today*, *if you are found abusing drugs*, *you are most likely to end up in police custody and you are likely to end in the hospital*. *A suicide attempt is also likely to end you in police custody*, *so that is how much the gap we have in terms of mental health services in Uganda”* (K12).

In one focus group, participants highlighted that most Ugandan men tended to hold adverse attitudes towards mental health and displayed a reluctance to be associated with such matters, often leading to blame and responsibility placed on women for children’s struggles. One participant remarked, *“Mainly men do not want to associate themselves with any issue related to mental illness and they tend to run away from the family; that’s why we have very many broken families”* (MC2). This results in some families isolating children with mental health concerns as opposed to help-seeking.

Professional stigma also hinders community mental health. As one key informant shared from their experience, *“when I was going to train for my diploma in mental health*, *my close friend asked me what are you going to be doing*, *how many madmen do you see on the streets that you will be able to help*. *They are just 2 or 3 and that’s all you will be doing in life* (K12).” Negative stereotypes permeate from even outside of the mental health profession. As one participant shared, *“there are instances where the president came out publicly and said we don’t need psychologists*, *and I think it is about the people who advise him*. *So*, *we need the right people to advise him*. *So we need people in parliament to lobby for mental health”* (K13).

#### Limited family engagement

Many participants recognized the value of relational family-based mental health models to progress community mental health needs outside of existing psychiatric models. These are therapy models that include family members or significant others in some way in treatment, either as direct support, or as a part of the problem resolution. With this acknowledgment, key informants described how limited training in systemic family therapy and family engagement skills impedes current efforts and contributes to the significant burden on the psychiatric care system. For example, if an adolescent was experiencing difficulties, not including caregivers or parents directly into treatment would add challenges when the individual returned home.

### System recommendations

Participants’ recommendations for strengthening the mental health system in Uganda included strategies to build on existing capacities, enhance education and training, implement systemic and integrated approaches, and advocate for policy and funding changes.

#### Enhance education and training

Participants identified continuing mental health education and training, and notably family therapy and community mental health trainings, as strategies to increase existing capacity. Family-level engagement, assessments, and psychoeducation were discussed as enhancements to service delivery as many service users may benefit from systemic therapy and intervention rather than, or in addition to medication support. One participant expressed, *“There is a gap*, *how I wish we meet the families*, *have a discussion maybe these issues will go away* (MC2).” Group counseling training was also notably discussed. Participants in one focus group shared: *“continuous education in group counseling is something we need to learn before starting to practice* (MC2).” Another discussed telehealth training; *“I know we have it but students have not yet appreciated tele-counseling*, *people have these beliefs that I must meet physically*…*so tele-counseling should also be advanced… sometimes they get used [to it]*. *For example*, *if someone has stigma and when we do tele-counseling*, *they realize that this is something good and they can come to the center after”* (MC2).

Further, participants shared that additional education on mental health conditions, relational effects, systemic approaches, and coping strategies would enhance the ability to respond to common, prevalent, and unaddressed needs related to depression, anxiety, substance use, trauma, caregiving stress, and relational violence. Participants also underscored community sensitization as a strategic effort that has demonstrated progress in enhancing community knowledge and reducing stigma around common mental health concerns. One participant shared knowledge of the approach, *“*…*we reach out to the communities at their location*, *that is their local villages*, *and we sensitize them*. *[We] work through their leaderships*, *their stakeholders at the community level*…*we work with them to identify places where we go and meet the people that have a need and we first give them information about mental health*, *and they actually get to realize that these things they have actually been suffering in the community are mental health conditions*. *So*, *then they come in for screening*, *we screen them* (K12).” Altogether, when delivered in professional, clinical, and community settings, education and training on family and community models was discussed as key source of mental health capacity building when delivered in professional and community settings.

#### Systemic and integrated approaches

Key informants shared the need for an integrated referral system. Suggestions focused on integrating providers that are available and trained in systemic-family therapy, community mental health models, social work, and other psychosocial intervention approaches. Many called for greater collaboration among providers with various training to enhance holistic responses to common community mental health needs. Traditional healers were frequently discussed by key informants due to the high likelihood of herbalists, traditionalists, and spiritual leaders being the first source of support community members seek to resolve personal and interpersonal issues. Key informants acknowledged that stigma would need to be addressed in order to successfully integrate and promote collaboration with traditional healers, many of whom are not included as referral sources of formal collaborators in the mental health system. Key informants also recommended that efforts shift toward greater focus on disability, religion, poverty, gender, and other social intersections that influence family and community mental health experiences.

#### Policy and advocacy

Participants agreed that policy advocacy was necessary enhance the Ugandan mental health system. Funding was most frequently discussed. As illustrated by a stakeholder, *“More funding*, *more resources to put mental health in the spotlight*. *I can give an example with HIV/ AIDS the government put a lot of effort*, *so*, *if they can put the same effort towards mental health*, *we would be in a better place* (K11).” Participants emphasized a pressing need to augment the budget allocated to mental health services. One participant articulated this sentiment, stating: *“mental health services get about 1% of the portion that is given to the health sector*. *That is really so little and it gives us a picture that mental health services have not been prioritized in Uganda*. *And yet*, *recently the statistics that were all over the media are that over 14 million Ugandans have been mentally ill*…*”* (K9).

While recognizing that national funding constraints limit the current distribution of resources in the mental health system, key informants equally recognized that lobbying for increased funding would help strengthen the foundation for an integrated referral system. Key informants shared that advocacy must also focus on creating employment pathways for professionals trained in family and community mental health to work within nationally funded healthcare centers. In the words of another, funding would also increase the potential for training and continuing education opportunities on systemic-relational approaches.

## Discussion

The goal of this study was to assess key stakeholders’ perceptions of the mental health system in Uganda; particularly their observations of strengths, challenges, and recommendations for improving capacity in the national system. This is important given the high rates of mental health difficulties in Uganda, and exacerbation of these difficulties by the COVID-19 pandemic and the high number of refugees present in Uganda. Stakeholders not only described their mental health system experiences engaging in service delivery, training, clinical supervision, research, and policy efforts to enhance mental healthcare, but they also offered critical perspectives on strategies to enhance capacity around existing efforts to address some of the most common mental health problems experienced by individuals, families, and communities in local Ugandan settings. They emphasized leveraging existing assets for sustainability, highlighting strengths like the mental health policy and referral system, despite resource constraints. However, barriers such as funding and stigma hindered efforts, underscoring the need to address socioeconomic and structural limitations to improve mental healthcare delivery in Uganda.

In terms of strengths, participants described a number of initiatives and realities that made mental health treatment access a reality for many Ugandans. High on the list of strengths is that the Ugandan government is aware of mental health prevalence and is committed to lowering mental health challenges in the country. Even though some pointed out that mental health care represents only a small percentage of the overall budget, and is small in comparison to the healthcare budget, there is, in policy, a commitment to alleviating mental health challenges. This is well reflected in the low costs of treatment for those who seek it, and free psychotropic medications provided. In addition, the mental health system is supported by a private sector (e.g., NGOs, private hospitals and health centers) that is committed to optimal mental health services, resources, and collaborations between researchers, clinicians, and the government. These joint efforts are essential in working to solve difficult problems. These strengths have contributed to increased awareness across Uganda of mental health concerns, possible treatments, and has worked to reduce stigma even though much work remains. In this sense, even though there is a shortage of mental health providers in Uganda (as in other African contexts), Uganda has important mental health initiatives underway. Despite these initiatives, Uganda still lags in World Health Organization ratings, ranking at 119 out of 190 nations for its health systems.

While Uganda assented to the Abuja declaration alongside other African governments that pledged to raise healthcare funding to 15% of the total budget in 2001, mental health remains a small part of this budget, and overall growth has been slower than expected in Uganda [33]. In this regard, it is imperative that mental healthcare capacity building efforts leverage existing resources. Engaging the existing strengths in a system enables sustainability, a critical capacity building outcome. Stakeholders described various assets and resources that currently contribute to national mental health efforts, while describing a range of opportunities to optimize the use of such assets. Participants largely endorsed the existing mental health policy and mental health system as a strength, despite resource limitations and staffing constraints.

While numerous strengths were reported, significant barriers and challenges to mental health care persist in Uganda. Participants’ reflections of challenges revealed the significant role of socioeconomic and structural limitations (i.e., funding, human resource limitations) on mental health care efforts in Uganda. Stakeholders unanimously acknowledge the constrained resources that have impeded the delivery of adequate services to Ugandans grappling with mental health difficulties. This necessitates greater attention from parliament, which holds the key to resource allocation. There has been progress in this regard in recent years. For example, on the 27th of September 2022, the Parliament of Uganda adopted a motion to urge the government to urgently increase the budget allocation to mental health and improvement of mental health services (Parliament of the Republic of Uganda, n.d.). It is ideal if the Ministry of Health’s budget is augmented, with a direct impact on boosting the mental health budget. This is especially crucial when it comes to continued increases of awareness of mental health concerns, reduction of stigma, and increasing accessibility to treatment. Stigma appears to still play a substantial role in hindering mental health care efforts in spite of progress, and this stigma as we show in this study, occurs at both the community and the professional levels.

A similar study that involved service users from Kampala and Kamuli in Eastern Uganda showed many of the same deficits in the mental health system. While Mugisha and colleagues’ [34] study revealed that service users were not as knowledgeable on policy, service users in our study were knowledgeable about the Ugandan Mental Health Act, its revisions, and the nature of its predominantly medical model. Despite this knowledge, service users felt that their voices were marginalized in the grand scheme of Ugandan healthcare.

While our findings are similar to Mugisha and colleagues’ [34] study engaging service users, differential results could be due to data collection timing (i.e., 3–4 years apart) or contextual differences (between Kampala and Kamuli, and Kampala and Gulu in the present study setting). Gulu is located in northern Uganda, a region affected by more than a decade of civil war with consequentially high prevalence rates of PTSD. Given that both governmental and NGO partners and peer support groups have had more time to develop community mental health programs in northern Uganda, awareness of mental health and peer support networks may differ in the northern region as compared to the urban and highly populated central region.

Feedback from mental health service users in this study underscores a crucial aspect often overlooked in LMIC settings. Many individuals facing mental health challenges emphasize that their issues do not always demand medicinal solutions. However, a prevalent obstacle remains—the difficulty in accessing mental health service providers who offer non-pharmacological interventions. This appears to be because of the lack of human resources with the time available to meet with individuals/families over a period of time. This was especially true when it came to considering treatments with strong evidence for their effectiveness in other contexts, treatments that engage members of the family, or other evidence-based approaches. There is still work to be done to move mental health treatments beyond medication prescribing and management to talk therapy approaches proven to work including evidence-based individual therapies, group therapies, and family-based treatments.

When reflecting on changes that could enhance the quality of services, training and education were frequently requested. Participants were interested in training in systemic and relational approaches. In addition, participants were interested in greater advocacy at the policy level that would involve a more aggressive approach to mental health. This would include more funding for mental health treatments as well as more training for mental health workers, essentially creating more workforce opportunities in mental health care.

In this study, participants discussed a dearth of evidence-based family interventions contrary to a systematic review conducted by Asiimwe et al. [35] that found that the existence of family and parenting evidence-based interventions in over 6 African countries including Uganda, were a unique strength. The reason for this discrepancy might be that the implementation of these evidence-based family interventions are mostly in targeted communities that have been reached by researchers and NGOs coming from outside Uganda, and they are not systematically rolled out to the public domain nationally through mechanisms facilitated by the ministry of health. While these types of systemic interventions are frequently mentioned in the literature and have promise of effectiveness, they were not experienced by our participants.

A noteworthy observation is the inclination of mental health service providers towards Western methods, sidelining the role of traditional healers or local culture and customs in treatment. One thing that stood out is that service recipients often resort to mental health hospitals as a last option, having exhausted traditional avenues. However, this is often after they have wrestled with the problem for a long time and tried different “home remedies.” Once they have received treatment at the hospital, a significant number continue seeking services from traditional healers. Bridging this gap is paramount, especially when it comes to aligning the approaches of mental health professionals and traditional healers. Sensitizing policymakers and service providers on the inextricable role of traditional healers in people’s lives could erode the existing divide. Education and mutual understanding may lead to an improved referral system, fostering collaboration between these two pillars of mental health support. Our findings align with research on sociocultural considerations of mental health in Uganda and support the idea that many Ugandans deeply value their culture and spiritual beliefs, thus a comprehensive mental health strategy would adequately attune to culture and context [34,35]. Using decontextualized care would undermine community mental health efforts and progress.

### Clinical implications

The implications drawn from the study emphasize the need for an integrated approach beyond medications, integrating non-psychiatric mental health professionals, engaging spiritual leaders and traditional healers, forming partnerships with NGOs, and enhancing training and supervision. This section delves into the clinical implications derived from the study, highlighting their significance in transforming the mental health landscape in Uganda.

Based on this study, we advocate for more comprehensive mental health approaches in Uganda. Mental health is a multifaceted concern, and the integration of non-psychiatric mental health professionals is crucial. It incorporates clinical psychologists, counseling psychologists, social workers, and family counselors into the mental health system. By doing so, the system can offer a comprehensive range of interventions, addressing psychological, social, and familial aspects of mental health beyond traditional medication-focused approaches.

Creating a collaborative model that integrates various mental health professionals is vital for a comprehensive and effective mental healthcare system. Establishing frameworks and training programs that foster teamwork between psychiatrists, psychologists, social workers, family counselors, and other professionals can lead to more cohesive and holistic approaches. Diverse skills and perspectives within the integrated team ensures a nuanced understanding of mental health issues and facilitates tailored interventions for individuals.

This study highlights cultural contexts of mental healthcare in Uganda and advocates for the engagement of spiritual and traditional healers [35]. To bridge the gap between modern mental health practices and traditional healing methods, cultural competency training for mental health professionals is essential. By establishing communication channels and collaborative platforms between spiritual leaders, traditional healers and mental health professionals, they can enhance the accessibility and acceptability of mental healthcare services among the population. Furthermore, acknowledging the role of non-governmental organizations (NGOs) in providing psychosocial services, the study suggests formal partnerships between governmental and non-governmental entities. These partnerships can lead to collaborative projects and initiatives, pooling resources, sharing expertise, and implementing best practices. By harnessing the strengths of both sectors, the mental health system in Uganda can be fortified, expanding its reach and impact on the community.

Moreover, the study underscores the importance of continuous professional development through additional training programs. Focused on the assessment and intervention of common community mental health problems such as depression, anxiety, and PTSD, these training modules can enhance the skills of mental health professionals. This investment in training ensures a workforce that is well-equipped to address prevalent mental health issues in the Ugandan community effectively.

The results of this study suggests that strengthening supervision processes and integrating systemic and community mental health skills can reinforce mental health system strengths. Regular supervision sessions emphasizing community-oriented approaches and family engagement skills training can empower the existing mental health workforce. This, in turn, can enhance family and community mental health outcomes, as well as relational support and wellbeing. The study’s clinical implications on the Ugandan national mental health system offer a roadmap for transformative change. By embracing a holistic approach, integrating diverse professionals, engaging spiritual leaders and traditional healers, forming partnerships, and investing in training and supervision, Uganda could build a mental healthcare system that is responsive, culturally sensitive, and effective. When implemented thoughtfully, these recommendations can improve mental health outcomes and contribute to the overall well-being of the Ugandan population.

### Research implications

One crucial research implication is the call for increased opportunities for research partnerships. By fostering collaborations between researchers, governmental bodies, and non-governmental organizations, prevalence studies can be conducted to understand better the diverse landscape of mental health challenges across different regions. This collaborative approach ensures a more comprehensive and nuanced understanding of mental health issues, facilitating the development of targeted interventions tailored to the unique needs of each region.

The study advocates for a shift in research focus from Western-centric models to an exploration of mental health experiences within the sociocultural context of Uganda. This implies engaging cultural advisors in research endeavors to provide insights into the cultural nuances that influence mental health perceptions, experiences, and help-seeking behaviors. By adopting a culturally sensitive approach, research can contribute to a more inclusive and representative understanding of mental health in Uganda.

Another significant implication is the call to increase mental health research in remote areas. Research should include underrepresented and underserved regions, ensuring findings reflect geographical diversity. This geographic diversification promotes a more equitable distribution of mental health research, uncovering challenges and opportunities unique to rural and remote communities and addressing disparities in mental healthcare access.

To increase inclusivity in mental health research, the study emphasizes the importance of engaging service users. Research should actively involve individuals who navigate mental health resources from diverse backgrounds, considering age, gender, socioeconomic status, and cultural heritage. An inclusive approach ensures that research findings reflect the experiences and perspectives of underrepresented stakeholders, fostering a deeper understanding of mental health experiences, interactions, and challenges across different demographic groups.

Recognizing the influence of traditional healing practices in Uganda, the study advocates for the inclusion of spiritual, religious, and traditional healers in mental health research. Research endeavors should seek collaboration with spiritual leaders and traditional healers to understand their perspectives, practices, and role in the mental health landscape. This collaboration can bridge the gap between traditional values and modern mental health practices, fostering cultural sensitivity in mental health research and professional practice. By developing research partnerships, embracing sociocultural perspectives, reaching beyond urban centers, engaging underrepresented service users, and involving traditional healers, future research endeavors can contribute significantly to a more comprehensive understanding of mental health capacity building in Uganda. This approach enriches the research landscape and lays the foundation for more effective and culturally responsive mental health interventions.

### Policy implications

A paramount policy implication of the study is the urgent need for increased funding and financial resources dedicated to mental health. Policy advocates should actively engage with government bodies, international organizations, and stakeholders to underscore the importance of allocating substantial financial resources to mental health initiatives. Adequate funding is essential for the implementation of comprehensive mental health programs, the establishment of community services, and the development of sustainable interventions. Policymakers must recognize the value of investing in mental health to improve public health and well-being.

The current study emphasizes the importance of decentralized resources to strengthen mental health services. Policies should promote equitable distribution of resources at the district level, including personnel, infrastructure, and funding. This decentralization fosters accessibility to mental health services in remote areas, addressing regional disparities and promoting community-based interventions. Policies advocating for resource allocation at the lower district levels are crucial for building a resilient and responsive mental health infrastructure.

Effective mental health policies require research-policy collaborations [36]. Advocacy efforts should focus on establishing partnerships that facilitate the translation of research findings into actionable policies. Policymakers should be engaged in research, providing insights into policy needs and challenges. Collaboration with advocacy organizations enhances the visibility and impact of research, contributing to developing evidence-based policies. Policies that integrate research findings into mental health frameworks are essential for creating compelling, responsive, and culturally sensitive mental health services.

A significant policy implication highlighted by the study is the necessity to promote collaboration in referral systems. Policies should be developed to encourage and regulate collaborative efforts between spiritual leaders, traditional healers, hospitals, and other professionals trained in mental health interventions. Advocacy efforts should focus on breaking down barriers between traditional and modern mental health practices, fostering an integrated approach. Policies promoting a harmonious referral system are essential for creating guidelines, protocols, and standards that ensure effective and culturally sensitive mental health care.

The policy implications derived from the study on the Ugandan national mental health system underscore the imperative for advocacy, collaboration, and inclusivity. Advocating for increased funding, decentralized resource allocation, collaboration with policymakers, and promoting a harmonious referral system are crucial steps toward building robust mental health policies. These policies, rooted in research and developed in collaboration with key stakeholders, will contribute to a more resilient, accessible, and culturally sensitive mental health system in Uganda. As policymakers embrace these recommendations, they have the potential to effect transformative change, enhancing the mental health and well-being of the Ugandan population.

### Limitations

A few limitations should be noted and considered in future studies to build on existing knowledge about mental health capacity building in Uganda. Prevalence studies continue to be important in order to map the diverse mental health issues affecting Ugandans, as well as the resources available to address common community mental health concerns. Prevalence studies can inform stakeholders on the areas that demand focused attention. Additionally, since the current study was limited to Kampala and Gulu, gathering perspectives from mental health service providers in other regions of Uganda is imperative to comprehensively understand the nation’s progress in delivering mental health services.

## Conclusions

The current ethnographic assessment of the Ugandan national mental health system offers a rich tapestry of insights into the strengths, challenges, and opportunities inherent in the current landscape. The study serves as a compass for navigating the complexities of mental health care in Uganda, providing a foundation for transformative changes. System strengths, including the resilience and dedication of mental health professionals, lay the groundwork for building upon existing positive aspects. However, the study also illuminates challenges that necessitate targeted interventions and policy adjustments. The call for increased funding and financial resources emerges as a crucial aspect of the study’s implications. Recognizing the importance of adequate financial support for mental health initiatives, the study advocates for reevaluating resource allocation, particularly emphasizing decentralized distribution to the lower district levels. This shift is essential to bridge regional disparities, ensuring that mental health services are accessible and responsive across diverse communities. Moreover, the study underscores the significance of collaborative efforts in mental health research and policy development.

The call to partner with policymakers, advocacy organizations, and traditional healers represents a recognition of the multi-faceted nature of mental health care. Collaboration amplifies the impact of research findings and fosters a more inclusive and culturally sensitive approach to mental health services. The results of this study emphasize collaboration between hospitals and clinics, NGOs and other mental health workers as a pivotal recommendation, recognizing the importance of cultural context in mental healthcare.

This study advances efforts in Uganda’s mental health system. Its comprehensive analysis illuminates the path forward, urging stakeholders to advocate for increased funding, decentralize resource allocation, foster collaborative research, and promote a harmonious referral system. The recommendations provided are poised to address existing challenges and advance opportunities for a more resilient, accessible, and culturally sensitive mental health system that caters to the diverse needs of the Ugandan population. The study, therefore, stands as a roadmap for shaping policies and practices that prioritize mental health and well-being in Uganda.

## Supporting information

S1 Checklist (DOCX)
