# Supplementary material for: Voices from the ground: Qualitative perspectives on Ugandan national mental health system strengths, challenges, and recommendations
Source: PLOS Ment Health. 2024 Nov 12;1(6):e0000077. doi: 10.1371/journal.pmen.0000077 (PMC12798241; doi:10.1371/journal.pmen.0000077)
Supplement: S1 Checklist — (DOCX) [file pmen.0000077.s001.docx]

Inclusivity in global research

PLOS’ policy on inclusivity in global research aims to improve transparency in the reporting of research performed outside of researchers’ own country or community and ensures that PLOS publications reporting global research adhere to high standards for research ethics and authorship. Authors of relevant research articles may be asked to complete the questionnaire below, which outlines ethical, cultural, and scientific considerations specific to inclusivity in global research. This questionnaire may be requested when researchers have travelled to a different country to conduct research, if research uses samples collected in another country, research with Indigenous populations or their lands, or if research is on cultural artefacts. Researchers travelling to another country solely to use laboratory equipment will not normally be required to complete the questionnaire. However, the questionnaire can be requested at the journal’s discretion for any submission – if you have been requested to complete this questionnaire by the PLOS journal you submitted to, please do so.

Please complete the questionnaire below and include this as a Supporting Information file with your manuscript. Note that if your paper is accepted for publication, this checklist will be published with your article in the supporting information files. Please ensure that you reference the checklist in the main body of your manuscript. We suggest adding a subsection ‘Inclusivity in global research’ to your Methods section and adding the following sentence: “Additional information regarding the ethical, cultural, and scientific considerations specific to inclusivity in global research is included in the Supporting Information (SX Checklist)”

The questions have been designed to be applicable to a wide range of study types, and there are subsections for both human subjects research and non-human subjects research. If any of the questions are not relevant to your research please mark them as “N/A” as appropriate.

**Ethical considerations, permits and authorship**

*This section is applicable to all research types.*

Provide details as to who granted permissions and/or consent for the study to take place in the Methods section of your manuscript. This should include the names of **all** ethics boards, governmental organizations, community leaders or other bodies that provided approval for the study. If individuals provided approval refer to these people by their role or title but do not list their name(s).
More information: This research received ethical approval from the Ethics Committee of Michigan State University on April 1^st^, 2022, and a local Ethical Commitee of Makerere University College of Health sciences in May 2022. Informed consent was obtained for data collection and publication of data. All the data collected from the study participants was conducted in accordance with the ethical standards of the institutional research committee Consent for publication

Reported on page number: Page 8 (Data Collection Procedures) sub-section of Methods

If there were any deviations from the study protocol after approval was obtained please provide details of these changes in the Methods section of your manuscript.
Did this study involve local collaborators that are residents of the country where the research was conducted or members of the community studied? If you do not have any authors from said communities, please provide an explanation for this below.

Reported on page number: Non-applicable (No deviations from protocol)

Yes, the second author and co-Principal Investigator of the current study is a resident of Uganda, and a leading professional in clinical psychology in the country. This collaborator is the corresponding author, Rosco Kasujja, [**rosco.kasujja@mak.ac.ug**](mailto:rosokasug@gmail.com) or [**rosokasug@gmail.com**](mailto:rosokasug@gmail.com)

Everyone listed as an author should meet PLOS’ criteria for authorship and all individuals who meet these criteria should be included in the author byline, rather than the acknowledgements. For further information please see the journal’s Authorship Policy.

**Human subjects research (e.g. health research, medical research, cross-cultural psychology)**

Did you obtain written informed consent from a representative of the local community or region before the research took place? How did you establish who speaks for the community? Details of written informed consent obtained from study participants should be reported separately in the Methods section of your manuscript.

We obtained written informed consent and signatures from stakeholders who engaged in key informant interviews and local focus groups. The IRB protocol for the current study was deemed “exempt” in status from both higher education institutions (in the U.S. and Uganda), but we still documented consent.

How did members of the local community provide input on the aims of the research investigation, its methodology, and its anticipated outcome(s)?

The project was developed, planned, and executed in collaboration with local professionals at Makerere University and worked together to establish culturally and locally-relevant aims for 9 months, prior to local data collection efforts

When engaging with the local community, how did you ensure that the informed consent documents and other materials could be understood by local stakeholders?

We developed all documents in English, which is commonly spoken among stakeholders in the current study, all of whom were fluent in English. We developed interview questions collaboratively, and held reflexive meetings to process cultural relevance of questions before and during data collection. Ugandan professionals were involved in the project development as key collaborators (co-PI, graduate research assistants). Focus groups were co-facilitated with Ugandan and U.S. based research team members.

Will the findings of the research be made available in an understandable format to stakeholders in the community where the study was conducted (e.g. via a presentation, summary report, copies of publications, etc.)? Please provide details of how this will be achieved.

A summary of findings and visual presentation of findings are developed for presentations understandable to local stakeholders. A recent opportunity has emerged to present key findings at Makerere University during an Alliance for African Partnership research colloquium in Fall 2024.

**Non-human subjects research using specimens/ animals collected as part of the study, or those housed in archival collections. Examples include archaeology, paleontology, botany and zoology.**

Did the permission you obtained from a local authority to perform the study include an agreement on access to outputs and benefit sharing? This may include procedures to enable fair distribution of the benefits and resources arising from the research performed. Please include any details of Prior Informed Consent and Benefit Sharing Agreements obtained. These may be required by field-specific regulations, for example the Convention on Biological Diversity (CBD) and the associated Nagoya Protocol.

Non-applicable

If the material used in your study was imported, please A) provide the year it was imported and B) indicate whether permits were obtained to import/export the materials used, C) provide details of any permits obtained. If this information is not available, please indicate this.

Non-applicable

If you used archival specimens, please state how the material used in your study was acquired by the institute it is held in and provide details of any permits obtained for the original excavations/ sample collection. If this information is not available, please indicate this.

Non-applicable

How was the potential cultural significance of the materials collected in your study to local communities considered in your research design? Were Indigenous peoples and/or local researchers and institutions involved with archaeological excavations / collection of specimens? If so, please provide a description of their involvement.

No specimens were collected. Local researchers and institutions contributed to culturally relevant methods from design to analyses, and are involved in collaborative manuscript development to disseminate the results.

If your manuscript includes photographs of human remains please indicate whether authors obtained permission from descendants or affiliated cultural communities to do so.

Non-applicable
